# Targeting lipocalin-2 in atherosclerosis: biological functions, pathogenic mechanisms, and therapeutic potential

**DOI:** 10.3389/fcvm.2026.1927210

**Published:** 2026-09-21

**Authors:** Zhixiang Gong, Haoyue Jia, Hao Zhang, Xiaohong Yan, Zhaoli Xiang, Qiang Wan

**Affiliations:** 1 Department of Critical Care Medicine, The Fourth Affiliated Hospital of Jiangxi University of Chinese Medicine, Nanchang, China; 2 Department of Critical Care Medicine, Jiangxi Provincial Hospital of Integrated Traditional Chinese and Western Medicine, Nanchang, China; 3 Clinical Medical College, Jiangxi University of Chinese Medicine, Nanchang, China; 4 Department of Medical Cardiology, Affiliated Hospital of Jiangxi University of Chinese Medicine, Nanchang, China

**Keywords:** atherosclerosis, foam cell, inflammation, lipid metabolism, lipocalin-2, therapeutic target

## Abstract

Atherosclerosis, the principal pathological underpinning of cardiovascular diseases, is a multifaceted process encompassing dysregulated lipid metabolism, chronic inflammation, oxidative stress, aberrant vascular smooth muscle cells (VSMCs) proliferation, and plaque destabilization. Lipocalin-2 (LCN-2), a pivotal member of the lipocalin superfamily, serves as a critical nexus integrating lipid metabolism with inflammatory cascades. This review systematically delineates the gene localization, protein architecture, and expression regulation of LCN-2, with a focus on its multifaceted roles and molecular mechanisms across diverse stages of atherosclerosis, including endothelial dysfunction, lipid deposition, inflammatory amplification, foam cell formation, VSMCs phenotypic switching, and plaque rupture. This work highlights the promise of LCN-2 as a diagnostic biomarker and therapeutic target, offering novel insights and strategic directions for the precise prevention and management of atherosclerosis. Given its pathogenic significance, elevated LCN-2 levels in serum, plasma, urine, and plaque tissue serve as diagnostic and prognostic biomarkers for atherosclerosis. Multiple therapeutic strategies targeting LCN-2, including gene knockout, neutralizing antibodies, small-molecule inhibitors, RNA interference and repurposed clinical drugs, have achieved encouraging anti-atherosclerotic effects in preclinical studies. Collectively, LCN-2 acts as a core molecular linker between inflammation and lipid metabolism in atherosclerosis, and targeting LCN-2 holds great potential for the early diagnosis, risk stratification and novel therapeutic intervention of atherosclerotic cardiovascular diseases.

## 1 Introduction

Cardiovascular diseases (CVDs) represent the foremost cause of mortality and disability worldwide, with an escalating prevalence that imposes a substantial burden on global healthcare systems (1). Atherosclerosis, the common pathological substrate of most CVDs including coronary artery disease, heart failure, and peripheral arterial disease, is a chronic, progressive inflammatory condition characterized by lipid accumulation within the arterial wall. Its pathogenesis involves a cascade of events driven by the interplay of diverse factors, cell types, and molecular pathways, culminating in the development of atherosclerotic plaques (2–4). While historically regarded as a lipid-centric disorder, the pivotal contribution of inflammation is now widely acknowledged. The “lipid–inflammation axis” hypothesis posits that inflammatory mechanisms are integral to all phases of atherosclerosis, from initiation and progression to plaque rupture and thrombosis (5). Consequently, identifying key molecular mediators that bridge lipid metabolism and inflammatory signaling has emerged as a central research focus.

Lipocalin-2 (LCN-2), also known as neutrophil gelatinase-associated lipocalin (NGAL), initially identified in 1993 within secondary granules of activated neutrophils, is a 25 kDa glycoprotein secreted under stress by a variety of cell types, including neutrophils, macrophages, endothelial cells, and adipocytes (6). Originally implicated solely in innate immunity and antibacterial defense, subsequent investigations have revealed its involvement in iron homeostasis, apoptosis, tumorigenesis, and metabolic syndrome (7). LCN-2 binds iron-chelating siderophores and small hydrophobic ligands, thereby modulating innate immunity, iron trafficking, and apoptotic processes (8). Notably, circulating and tissue LCN-2 levels are markedly elevated in patients with obesity, insulin resistance, diabetes, and CVDs, as well as in corresponding animal models, and correlate positively with disease severity (9, 10). A growing body of clinical and experimental evidence now underscores a strong association between LCN-2 and atherosclerosis. A clinical research result indicated that serum LCN-2 holds promise as a clinically useful biomarker for the detection of unstable carotid plaques among asymptomatic individuals, nearly twofold higher serum LCN-2 levels were detected in patients with American Heart Association type VI plaques compared to those with other plaque types (11). Moreover, Serum LCN-2 concentrations are significantly increased in atherosclerotic patients and positively correlate with the extent of coronary stenosis, carotid intima-media thickness, and the incidence of adverse cardiovascular events (12). In atherosclerotic animal models such as ApoE^−/−^ and LDLR^−/−^ mice, LCN-2 expression is up-regulated in lesioned arteries, whereas its genetic deletion or antibody-mediated neutralization markedly attenuates atherosclerotic lesion development and enhances plaque stability (13, 14). These observations collectively position LCN-2 as a key regulatory molecule in the pathogenesis of atherosclerosis, highlighting its potential as both a diagnostic biomarker and a therapeutic target.

This article aims to systematically synthesize current knowledge on the biological features of LCN-2, comprehensively elucidate its regulatory functions and molecular mechanisms across various stages of atherosclerosis, and provide an extensive overview of anti-atherosclerotic agents that modulate LCN-2 expression. This synthesis aims to serve as a valuable resource for advancing research on the mechanisms of atherosclerosis and for devising innovative preventive and therapeutic strategies.

## 2 Biological characteristics of LCN-2

### 2.1 Gene and protein structure of LCN-2

#### 2.1.1 Gene localization and architecture

The human LCN-2 gene is situated on chromosome 9 at region q34.11, while its murine counterpart resides on chromosome 2, the human gene spans approximately 5.6 kb and comprises 7 exons and 6 introns, its cDNA is about 1.1 kb in length, encoding a precursor protein of 178 amino acid residues (15). The promoter region is enriched with binding motifs for transcription factors such as nuclear factor-kappa B (NF-*κ*B), signal transducer and activator of transcription 3 (STAT3), and peroxisome proliferator-activated receptors (PPARs), providing a molecular foundation for its regulated expression (16).

#### 2.1.2 Protein structure and molecular forms

The LCN-2 precursor includes an N-terminal 20-amino acid signal peptide that directs its secretion. The mature glycoprotein exhibits a molecular mass of approximately 25–28 kDa, with variability attributable to glycosylation modifications (17). Crystallographic analyses reveal a highly conserved *β*-barrel structure formed by 8 antiparallel *β*-strands (A–H). The interior of this barrel constitutes a hydrophobic binding pocket that selectively accommodates small hydrophobic ligands such as siderophores, fatty acids, and retinol, forming the structural basis for LCN-2's diverse biological activities (18). *In vivo*, LCN-2 exists predominantly in two distinct forms, namely as a heterodimer with matrix metalloproteinase-9 (MMP-9), primarily found in neutrophils where it stabilizes MMP-9 and modulates its substrate specificity, and as a free homodimer or monomer widely distributed in serum, urine, and various tissue secretions, wherein it exerts its intrinsic biological functions autonomously (19, 20).

### 2.2 Expression and regulation of LCN-2

#### 2.2.1 Tissue distribution and cellular origins

Under physiological conditions, LCN-2 is constitutively expressed at low levels in various tissues, including neutrophils, liver, kidneys, adipose tissue, and lungs (21). Neutrophils represent the primary reservoir, releasing substantial quantities of LCN-2 via degranulation upon activation. The liver is the main source of circulating LCN-2, with hepatocytes exhibiting baseline expression that is further inducible under stimulation. Renal proximal tubular epithelial cells also express LCN-2 significantly, rendering it a diagnostic biomarker for acute kidney injury. Under pathological conditions, multiple cell types including endothelial cells, macrophages, vascular smooth muscle cells (VSMCs), adipocytes, and tumor cells, can be stimulated to highly express LCN-2. Its expression level closely mirrors the degree of inflammatory activation, oxidative stress, and metabolic dysregulation (22, 23).

#### 2.2.2 Regulatory mechanisms

As generalized in Figure 1, LCN-2 expression is precisely governed by a complex network of signaling cascades and transcription factors. Inflammatory cytokines serve as primary inducers, tumor necrosis factor-*α* (TNF-*α*) and interleukin-1*β* (IL-1*β*) activate the NF-*κ*B pathway, promoting nuclear translocation of the p65/p50 dimer and its subsequent binding to *κ*B motifs within the LCN-2 promoter, thereby enhancing transcription, while interleukin-6 (IL-6) activates the Janus kinase (JAK)/STAT3 axis, leading to phosphorylated STAT3 dimer translocation and binding to STAT3-responsive elements in the promoter region (16, 24, 25). Parallel to these inflammatory signals, oxidative stress pathways also contribute significantly. Reactive oxygen species (ROS), generated by stimuli such as oxidized low density lipoprotein (ox-LDL) and high glucose via NADPH oxidase and mitochondrial electron transport, activate mitogen-activated protein kinases (MAPKs) cascades including ERK1/2, JNK, and p38 MAPK, resulting in downstream induction of activator protein-1 (AP-1) which binds AP-1 sites in the LCN-2 promoter, while ROS can also indirectly activate NF-*κ*B through oxidative inactivation of its inhibitory proteins (26, 27). Metabolism-associated regulation involves PPAR*α*/*γ* agonists, which suppress LCN-2 transcription by binding PPAR response elements (PPREs) in the promoter, whereas free fatty acids (FFAs) up-regulate LCN-2 expression through protein kinase C (PKC) activation or PPAR inhibition, and under insulin-resistant conditions, impaired insulin signaling promotes LCN-2 expression in adipose tissue via NF-*κ*B activation (28). Additional regulatory layers include hypoxia-inducible factor-1*α* (HIF-1*α*)-mediated upregulation under low oxygen conditions, transforming growth factor-*β* (TGF-*β*)-induced suppression through the Smad pathway, and post-transcriptional inhibition by microRNAs such as miR-122 and miR-223, which target the 3′-UTR of LCN-2 mRNA (29, 30).

**Figure 1:**
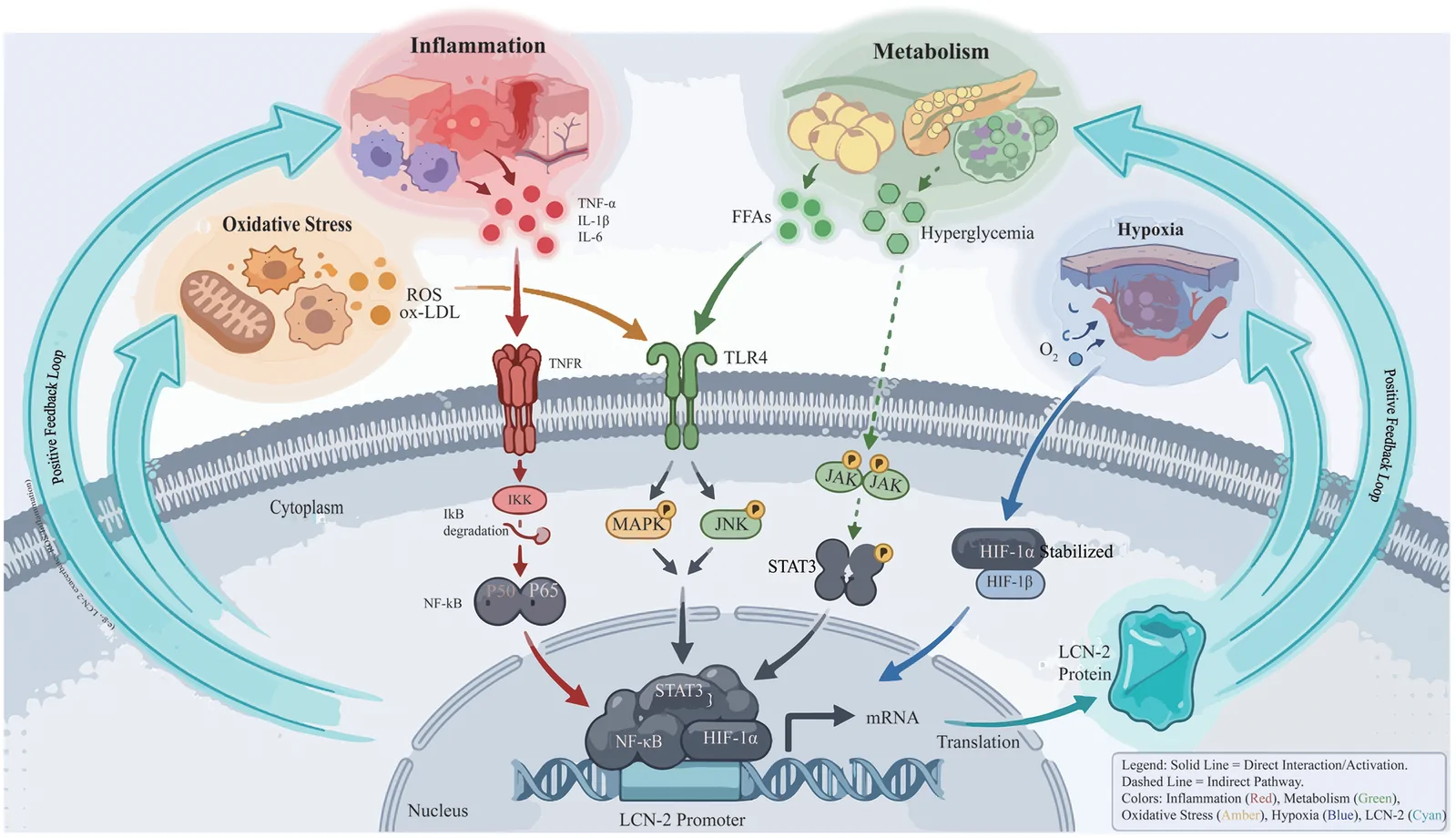
Major upstream signaling pathways governing LCN-2 transcription and expression.

### 2.3 Biological functions of LCN-2

#### 2.3.1 Modulation of inflammatory responses

LCN-2 functions as a critical immunomodulatory protein that orchestrates both pro-inflammatory and anti-inflammatory responses across various cell types and disease contexts. As an acute-phase protein, its expression is rapidly and robustly induced by inflammatory stimuli such as lipopolysaccharide, IL-1β and TNF-*α* through activation of NF-*κ*B signaling pathway (31). This places LCN-2 at the nexus of the innate immune system's response to stress, infection, and tissue injury.

The primary mechanism through which LCN-2 modulates inflammation involves its role as a chemotactic factor that directs immune cell migration. It has been shown to facilitate neutrophil recruitment to sites of inflammation and infection, thereby amplifying the local immune response (32). Additionally, LCN-2 influences macrophage polarization, promoting a shift toward the pro-inflammatory M1 phenotype while suppressing the anti-inflammatory M2 state (33). This regulatory capacity extends to other immune cells, including dendritic cells and T lymphocytes, where it affects their activation status and cytokine production profiles (34).

Interestingly, LCN-2 exhibits context-dependent dual functionality in inflammatory regulation. In acute inflammatory settings, it typically enhances the immune response to eliminate pathogens and clear cellular debris. However, in chronic inflammatory conditions such as obesity, atherosclerosis, and inflammatory brain disease, sustained elevation of LCN-2 can contribute to pathological inflammation and tissue damage (35). For instance, in adipose tissue of obese individuals, LCN-2 promotes macrophage infiltration and pro-inflammatory cytokine secretion, exacerbating insulin resistance (36). Conversely, in certain contexts such as acute kidney injury, LCN-2 may exert protective effects by limiting excessive inflammation and promoting tissue repair (37). Through these complex, context-specific actions, LCN-2 serves as a key rheostat of inflammatory processes, making it both a valuable biomarker for inflammatory diseases and a potential therapeutic target for modulating immune responses.

#### 2.3.2 Regulation of lipid metabolism

LCN-2 has emerged as a significant adipokine that modulates various aspects of lipid metabolism and energy homeostasis. LCN-2 is now understood to be highly expressed in adipose tissue and hepatocytes, where it functions as a key regulator of metabolic processes. Its expression is markedly up-regulated in obese states and correlates positively with body mass index, insulin resistance, and other metabolic parameters, suggesting its involvement in the pathophysiology of obesity-related disorders (38).

One of the primary mechanisms by which LCN-2 influences lipid metabolism is through its interaction with established metabolic pathways. Studies have demonstrated that LCN-2 can bind to and transport small lipophilic substances, potentially including fatty acids and other lipid moieties, thereby affecting their distribution and cellular uptake (39). Furthermore, LCN-2 has been shown to modulate adipocyte function by influencing the expression of PPAR*γ*, a master regulator of adipogenesis and lipid storage. This interaction can alter the differentiation capacity of preadipocytes and affect the overall lipid accumulation within adipose tissue (40).

In the liver, LCN-2 contributes to the regulation of lipid homeostasis by influencing hepatic fatty acid oxidation and triglyceride accumulation. Experimental evidence indicates that LCN-2 deficiency can lead to altered lipid profiles, including changes in circulating free fatty acids and cholesterol levels (41). Additionally, LCN-2 appears to play a protective role against hepatic steatosis by modulating inflammatory responses within the liver, thereby indirectly affecting lipid deposition (42). Through these multifaceted actions in adipose tissue and the liver, LCN-2 serves as an important molecular link between obesity, inflammation, and metabolic dysregulation, making it a potential therapeutic target for metabolic diseases such as type 2 diabetes and non-alcoholic fatty liver disease.

## 3 Regulatory role of LCN-2 in atherosclerotic development

Clinical Studies have provided evidence that LCN-2 can serve as a diagnostic biomarker for atherosclerosis. In serum, LCN-2 concentrations are significantly elevated in patients with atherosclerosis, and this increase shows a positive correlation with the severity of coronary artery stenosis, carotid intima-media thickness (CIMT), and the risk of major adverse cardiovascular events (MACE) (43). Consequently, serum LCN-2 is considered to possess high diagnostic and prognostic utility. Similar findings are observed in plasma, where LCN-2 levels are also markedly increased and correlate well with established inflammatory and oxidative stress markers, including high-sensitivity C-reactive protein (hs-CRP), IL-6, and ox-LDL (44). The diagnostic and prognostic value of plasma LCN-2 is likewise rated as high, underscoring its potential as a readily accessible circulatory biomarker. Urinary LCN-2 shows a moderate elevation in atherosclerotic patients, and its levels are associated with renal function parameters and overall vascular risk. Although the changes are less pronounced than in blood, urinary LCN-2 still offers moderate diagnostic value, which may be particularly useful in specific clinical settings where non-invasive sampling is preferred or where renal involvement is suspected (45). The most striking alteration is observed within the plaque tissue itself, where LCN-2 expression is markedly increased. This tissue-level elevation correlates strongly with plaque vulnerability and local inflammatory activity, thereby providing high diagnostic and prognostic value that is tissue-specific (46, 47). Collectively, LCN-2 is a versatile biomarker whose diagnostic performance varies depending on the sample type. Circulating LCN-2 offers robust systemic assessment, while urinary LCN-2 provides moderate but complementary information, and plaque tissue LCN-2 delivers the strongest lesion-specific readout. These multi-compartment findings support the integration of LCN-2 measurement into clinical strategies for risk stratification, early detection, and monitoring of atherosclerotic burden and plaque instability, as generalized in Table 1.

**Table 1:** Clinical evidence of LCN-2 as a diagnostic biomarker for atherosclerosis.

Sample Type	Change in AS patients	Correlation Index	Diagnostic/Prognostic Value	Ref.
Serum	Significantly increased	Coronary stenosis, CIMT, MACE risk	High	(43)
Plasma	Significantly increased	hs-CRP, IL-6, ox-LDL	High	(44)
Urine	Moderately increased	Renal function, vascular risk	Moderate	(45)
Plaque tissue	Markedly increased	Plaque vulnerability, inflammation	High (tissue-specific)	(46, 47)

### 3.1 LCN-2 and endothelial dysfunction

#### 3.1.1 Impairment of nitric oxide bioavailability

LCN-2 contributes to endothelial dysfunction through multiple mechanisms that converge on the impairment of nitric oxide (NO) bioavailability, a hallmark of vascular pathology. As a critical adipokine and inflammatory mediator, elevated LCN-2 levels in conditions such as obesity, diabetes, and metabolic syndrome are closely associated with endothelial activation and subsequent vascular damage.

The primary mechanism by which LCN-2 compromises NO bioavailability involves the induction of oxidative stress within the endothelial microenvironment. LCN-2 promotes the generation of ROS by activating NADPH oxidases and by disrupting intracellular iron homeostasis, as excess free iron can catalyze the formation of highly reactive hydroxyl radicals through Fenton chemistry (48). These ROS rapidly scavenge nitric oxide through a diffusion-limited reaction, forming peroxynitrite, a potent oxidant that further exacerbates endothelial injury. Peroxynitrite not only reduces the half-life and functional capacity of NO but also uncouples endothelial nitric oxide synthase (eNOS), shifting this enzyme from producing NO to generating additional superoxide anions, thereby creating a vicious cycle of oxidative stress and diminished NO availability in endothelial cells (49).

Furthermore, LCN-2-driven inflammatory signaling, particularly through Nrf2/HO-1 activation, can down-regulate eNOS expression and phosphorylation at key regulatory sites such as Ser1177, which is essential for optimal enzyme activity (50). The resultant reduction in NO synthesis, combined with accelerated NO degradation by ROS, impairs endothelium-dependent vasodilation, promotes platelet aggregation, and facilitates leukocyte adhesion (51). Through these oxidative and inflammatory pathways, LCN-2 critically undermines NO bioavailability, positioning it as a significant contributor to the pathogenesis of hypertension, atherosclerosis, and other cardiovascular complications associated with metabolic diseases.

#### 3.1.2 Induction of adhesion molecule and chemokine expression in endothelial cells

LCN-2 potently promotes endothelial dysfunction by directly inducing the expression of adhesion molecules and chemokines in endothelial cells, thereby facilitating vascular inflammation and the initiation of atherosclerotic lesions. As a pro-inflammatory adipokine elevated in obesity and metabolic syndrome, LCN-2 activates endothelial cells, transitioning them from a quiescent state to an activated phenotype that actively recruits immune cells (29). Mechanistically, LCN-2 binds to its receptor SLC22A17, also known as 24p3R, on the endothelial cell surface, triggering intracellular signaling cascades involving NF-*κ*B and MAPKs (52). These pathways drive the transcriptional up-regulation of key adhesion molecules, including vascular cell adhesion molecule-1 (VCAM-1), intercellular adhesion molecule-1 (ICAM-1), and E-selectin (53). The increased surface expression of these molecules enables the firm adhesion and rolling of circulating monocytes and neutrophils along the vascular endothelium, an essential early step in atherogenesis.

Concurrently, LCN-2 stimulates endothelial cells to produce and secrete potent chemokines, most notably monocyte chemoattractant protein-1 (MCP-1) and IL-8 (54). These chemokines establish a concentration gradient that directs the transmigration of adhered leukocytes into the subendothelial space. Once infiltrated, these immune cells propagate local inflammation, release cytokines, and contribute to the formation of foam cells, thereby accelerating plaque development (55). By orchestrating this coordinated up-regulation of both adhesion machinery and chemoattractant signals, LCN-2 acts as a critical mediator of endothelial activation and vascular inflammation. This mechanism directly links elevated LCN-2 levels to the pathogenesis of atherosclerosis and other cardiovascular complications associated with chronic inflammatory diseases.

#### 3.1.3 Disruption of endothelial barrier integrity

LCN-2 contributes significantly to endothelial dysfunction by disrupting the integrity of the endothelial barrier, a critical structure that maintains vascular homeostasis and prevents uncontrolled leakage of fluids and macromolecules into surrounding tissues (56). The endothelial barrier, maintained by junctional complexes including adherens junctions such as vascular endothelial-cadherin, and tight junctions such as claudins and occludins, serves as a selective filter between the circulation and the interstitium (57). Disruption of this barrier is a hallmark of vascular inflammation and a key initiating step in atherosclerosis.

Elevated LCN-2 levels directly compromise endothelial barrier function through multiple mechanisms. LCN-2 activates Rho GTPase signaling pathways, particularly RhoA, leading to increased actin stress fiber formation and actomyosin contraction. This cytoskeletal rearrangement generates intercellular gaps by exerting tensile forces on cell-cell junctions, thereby increasing endothelial permeability. Concurrently, LCN-2 promotes the phosphorylation and internalization of vascular endothelial-cadherin, a core component of adherens junctions essential for maintaining junctional integrity. Moreover, a clinical research has confirmed that LCN-2 promotes endothelial cell ferroptosis by regulating the HMGB1/Nrf2/HO-1 pathway, and aggravates the dysfunction of the endothelial barrier after intravenous thrombolysis (58). The loss of vascular endothelial-cadherin at intercellular contacts further destabilizes the barrier architecture. Additionally, the results of the experimental study indicated that LCN-2-driven inflammatory signaling stimulates the production of vascular endothelial growth factor (VEGF) and other permeability-inducing factors within the endothelial microenvironment (29). These mediators act synergistically to increase microvascular permeability, facilitating plasma leakage and leukocyte extravasation. The resulting edema and inflammatory cell infiltration exacerbate local tissue injury and perpetuate a cycle of vascular dysfunction. Through these coordinated actions on the endothelial cytoskeleton and intercellular junctions, LCN-2 undermines vascular barrier integrity, promoting a pro-permeable state that contributes to the pathogenesis of atherosclerosis.

### 3.2 Lipid deposition, and foam cell formation

#### 3.2.1 Facilitation of LDL oxidation

LCN-2 plays a pivotal role in atherosclerotic pathogenesis by facilitating the oxidative modification of LDL, a critical initial step that drives lipid deposition and foam cell formation within the arterial wall. The pro-oxidant activity of LCN-2 is intimately linked to its function as a modulator of iron homeostasis. By binding to bacterial siderophores, LCN-2 disrupts local iron trafficking, leading to the accumulation of labile iron within the vascular microenvironment (59). This labile iron serves as a potent catalyst for Fenton chemistry, generating hydroxyl radicals that directly initiate lipid peroxidation of LDL particles. Furthermore, LCN-2 promotes the activation of NADPH oxidases in endothelial cells and macrophages, amplifying the production of superoxide anions and other reactive oxygen species that further propagate the oxidative cascade (48). Ox-LDL generated through these LCN-2-dependent mechanisms is not merely a passive byproduct but an active driver of atherogenesis. Ox-LDL is rapidly taken up by macrophages via scavenger receptors such as CD36 and scavenger receptor class A (SR-A), bypassing the normal feedback regulation of cholesterol uptake and leading to unbridled intracellular cholesterol accumulation and the formation of lipid-laden foam cells (60, 61). Concurrently, LCN-2 up-regulates the expression of scavenger receptors on macrophages, further enhancing their capacity for ox-LDL internalization (62). Through promoting LDL oxidation while augmenting macrophage scavenger receptor expression, LCN-2 establishes a pro-atherogenic axis that accelerates lipid deposition and foam cell transformation, positioning it as a critical link between metabolic inflammation and the initiation of atherosclerotic lesions.

#### 3.2.2 Enhancement of ox-LDL uptake by macrophages

LCN-2 critically accelerates atherosclerotic progression by directly enhancing the uptake of ox-LDL via macrophages, a central mechanism driving foam cell formation and pathological lipid deposition within the arterial wall. Macrophages under inflammatory conditions exhibit markedly increased expression of LCN-2, which in turn acts in an autocrine and paracrine manner to amplify their scavenger function (63). Specifically, LCN-2 up-regulates the expression of key scavenger receptors, most notably CD36 and SR-A, on the macrophage surface. This up-regulation is mediated through activation of PPAR*γ* and downstream signaling cascades involving MAPKs, which transcriptionally enhance receptor synthesis (64). Consequently, LCN-2-primed macrophages display a heightened capacity for ox-LDL binding and internalization.

Moreover, LCN-2 suppresses the expression of cholesterol efflux transporters such as ATP-binding cassette (ABC) transporters, particularly ABCA1 and ABCG1, thereby impairing reverse cholesterol transport and promoting intracellular lipid retention (65). This dual effect of increased intake of oxidized low-density lipoproteins and decreased cholesterol excretion leads to a profound pro-atherogenic imbalance. The net result is unbridled accumulation of cholesteryl esters within macrophages, culminating in their transformation into foam cells, the hallmark of early atherosclerotic lesions. Additionally, LCN-2 can influence the polarization of macrophages toward a pro-inflammatory M1 phenotype, which further sustains scavenger receptor expression and amplifies the inflammatory milieu (66). Through this coordinated enhancement of ox-LDL uptake and inhibition of cholesterol clearance, LCN-2 serves as a pivotal mediator linking sterile inflammation to foam cell biogenesis and the initiation of atherosclerosis.

#### 3.2.3 Suppression of macrophage cholesterol efflux

LCN-2 contributes critically to foam cell formation and atherosclerotic lesion development by suppressing macrophage cholesterol efflux, thereby disrupting the delicate balance between lipid uptake and clearance that is essential for maintaining cellular cholesterol homeostasis (67). Cholesterol efflux, the process by which excess intracellular cholesterol is transported out of macrophages for eventual hepatic elimination, represents a protective mechanism against foam cell formation. This process is primarily mediated by ABCA1 and ABCG1, which facilitate the transfer of cholesterol to apolipoprotein A-I (apoA-I) and high-density lipoprotein (HDL), respectively (68).

The experimental research results indicated that LCN-2 can downregulate the expression of both ABCA1 and ABCG1 in macrophages through multiple interconnected mechanisms. Mechanistically, LCN-2 activates inflammatory signaling cascades, including the NF-*κ*B pathway, which in turn suppresses liver X receptor (LXR) activity, the master transcriptional regulator of ABC transporters. By inhibiting LXR and its downstream targets, LCN-2 effectively reduces the transcription of ABCA1 and ABCG1, impairing the macrophage's capacity to efflux cholesterol (69). Additionally, LCN-2 promotes the degradation of existing ABCA1 protein through post-translational modifications, further compromising efflux efficiency (70). The suppression of cholesterol efflux, when combined with the concurrent enhancement of ox-LDL uptake described earlier, creates a profound intracellular cholesterol accumulation that drives foam cell transformation.

Furthermore, LCN-2-mediated iron dysregulation can indirectly impair cholesterol efflux by promoting oxidative stress, which damages ABC transporters and reduces their functional activity (71). Through this coordinated suppression of reverse cholesterol transport pathways, LCN-2 establishes a pro-atherogenic intracellular milieu that favors progressive lipid deposition and foam cell formation, underscoring its significance as a key contributor to the pathogenesis of atherosclerosis.

### 3.3 LCN-2 and inflammatory amplification

#### 3.3.1 Control of macrophage polarization and activation

LCN-2 serves as a pivotal orchestrator of inflammatory amplification in atherosclerosis by exerting profound control over macrophage polarization and activation states (72). Within atherosclerotic lesions, macrophages exhibit remarkable phenotypic plasticity, broadly categorized into the pro-inflammatory M1 phenotype and the anti-inflammatory/pro-resolving M2 phenotype. The balance between these polar states critically determines lesion progression or regression. LCN-2, abundantly expressed in atherosclerotic plaques, skews this equilibrium decisively toward the M1-dominant state, thereby perpetuating and amplifying vascular inflammation.

LCN-2 activates the STAT1 and NF-*κ*B signaling pathways, both of which are master regulators of M1 polarization (73). This leads to the upregulation of classical M1 markers, including inducible nitric oxide synthase (iNOS), tumor necrosis factor-alpha (TNF-α), and interleukin-6 (IL-6). Concurrently, LCN-2 suppresses STAT3 and PPAR*γ* signaling, thereby inhibiting the acquisition of the M2 phenotype characterized by markers such as arginase-1, IL-10, and CD206 (41, 64, 74). The resulting M1-skewed macrophage population secretes an array of pro-inflammatory cytokines and chemokines that recruit additional immune cells to the lesion site, establishing a positive feedback loop of inflammatory amplification.

Furthermore, LCN-2 promotes metabolic reprogramming of macrophages toward aerobic glycolysis, a metabolic state that supports M1 activation and sustained pro-inflammatory cytokine production (7). This metabolic shift, reminiscent of the Warburg effect, further reinforces the inflammatory phenotype. By driving macrophage polarization toward the pro-inflammatory M1 phenotype while simultaneously restraining the anti-inflammatory M2 arm, LCN-2 acts as a critical node in the inflammatory cascade that fuels atherosclerotic progression, plaque instability, and the heightened inflammatory tone characteristic of advanced lesions.

#### 3.3.2 Modulation of neutrophil function and NETosis

LCN-2 acts as a potent modulator of neutrophil function and neutrophil extracellular trap (NET) formation, a process termed NETosis, thereby amplifying the inflammatory cascade that drives atherosclerosis progression (75). Neutrophils are among the first immune cells recruited to sites of vascular inflammation, and their activation state significantly influences plaque development and stability (76). LCN-2, which is abundantly secreted by activated neutrophils and other inflammatory cells within atherosclerotic lesions, functions as a chemotactic factor that promotes neutrophil recruitment to the arterial wall. Once recruited, LCN-2 primes neutrophils for enhanced activation and facilitates the induction of NETosis.

LCN-2 triggers NETosis through activation of NADPH oxidase and the subsequent generation of ROS, which are critical mediators of nuclear membrane disruption and chromatin decondensation (77). Additionally, LCN-2 can induce calcium flux and activate peptidylarginine deiminase 4 (PAD4), an enzyme essential for histone citrullination and NET formation. The resulting NETs, which are extracellular web-like structures composed of decondensed chromatin studded with granular enzymes, are released into the vascular microenvironment and exert multiple pro-atherogenic effects (78). NETs directly trap oxidized lipoproteins, promoting lipid deposition within the plaque, and activate endothelial cells, enhancing adhesion molecule expression and further immune cell recruitment. Moreover, NET components such as myeloperoxidase and neutrophil elastase degrade the fibrous cap, contributing to plaque instability and vulnerability to rupture (79).

Through this dual role in recruiting neutrophils and promoting NETosis, LCN-2 establishes a self-amplifying inflammatory loop in which NETs release additional pro-inflammatory mediators that recruit more neutrophils, while LCN-2 itself sustains the activated state of these cells. This amplification mechanism positions LCN-2 as a critical upstream regulator of neutrophil-driven inflammation in atherosclerosis, linking innate immune activation to plaque progression and adverse thrombotic complication.

#### 3.3.3 Regulation of lymphocyte activation and cytokine networks

LCN-2 amplifies the inflammatory milieu in atherosclerosis by exerting precise control over lymphocyte activation and the intricate cytokine networks that orchestrate adaptive immune responses within the plaque. While much attention has focused on its effects on innate immunity, accumulating evidence indicates that LCN-2 significantly influences T cell differentiation, activation, and effector functions, thereby contributing to the chronic inflammatory state characteristic of advanced atherosclerotic lesions (80).

LCN-2 modulates the balance between pro-inflammatory and regulatory T cell subsets. It promotes the differentiation of naïve CD4⁺ T cells toward the Th1 and Th17 lineages, both of which are implicated in atherosclerotic progression (81). This is achieved through activation of STAT1 and STAT3 signaling pathways, coupled with the induction of lineage-specific transcription factors such as T-bet and ROR*γ*t (82, 83). Th1 cells secrete interferon-gamma (IFN-*γ*), which activates macrophages and enhances foam cell formation, promoting neutrophil recruitment and vascular inflammation (84). Concurrently, LCN-2 suppresses the differentiation and expansion of regulatory T cells (Tregs), which normally serve a protective role by secreting anti-inflammatory cytokines such as IL-10 and transforming growth factor-*β* (TGF-*β*) (85). This disruption of Treg homeostasis further tips the balance toward a pro-inflammatory immune landscape.

Additionally, LCN-2 influences the activation of antigen-presenting cells, including dendritic cells, thereby shaping the cytokine microenvironment that directs T cell responses (86). It promotes the secretion of pro-inflammatory cytokines such as IL-6, IL-1β, and TNF-α, which not only sustain T cell activation but also establish positive feedback loops that amplify local inflammation. Through its integrated regulation of lymphocyte polarization and cytokine networks, LCN-2 functions as a critical bridge linking innate and adaptive immunity, perpetuating the inflammatory amplification that drives atherosclerosis progression and plaque destabilization.

### 3.4 LCN-2 and VSMCs abnormalities

Aberrant VSMCs proliferation, migration, and phenotypic transition are key events in fibrous plaque formation and plaque destabilization in atherosclerosis (87). Under physiological conditions, VSMCs maintain a contractile phenotype, essential for vascular structure and function. Under pathological stimuli, VSMCs switch to a synthetic phenotype, characterized by enhanced proliferative and migratory capacities and aberrant extracellular matrix (ECM) synthesis. LCN-2 regulates VSMCs dysfunction through multiple mechanisms.

#### 3.4.1 Induction of VSMCs phenotypic switching

LCN-2 contributes critically to atherosclerotic plaque formation and progression by inducing phenotypic switching of VSMCs, a process that transforms these cells from their quiescent, contractile state into a proliferative, synthetic phenotype that drives pathological vascular remodeling (88). Under physiological conditions, VSMCs maintain vascular tone and structural integrity through the expression of contractile markers such as *α*-smooth muscle actin (*α*-SMA), smooth muscle myosin heavy chain, and calponin. However, within the inflammatory microenvironment of atherosclerotic lesions, elevated LCN-2 acts as a potent trigger for VSMCs dedifferentiation.

Mechanistically, LCN-2 activates multiple signaling cascades that orchestrate phenotypic modulation. It stimulates the MAPKs pathway, particularly extracellular signal-regulated kinase (ERK) and p38, which promotes the down-regulation of contractile gene expression (89). Concurrently, LCN-2 activates NF-*κ*B signaling, inducing the expression of transcription factors such as Krüppel-like factor 4 (KLF4) and myocardin-related transcription factor A (MRTF-A), which drive the reprogramming toward a synthetic phenotype (90). This phenotypic switch is characterized by reduced expression of contractile proteins and up-regulation of synthetic markers including osteopontin, matrix metalloproteinases (MMPs), and various growth factors (91).

The synthetic VSMCs generated through LCN-2-mediated phenotypic switching exhibit heightened proliferative and migratory capacities, enabling them to migrate from the media into the intima, where they contribute to neointimal hyperplasia and fibrous cap formation (92). However, these dedifferentiated VSMCs also acquire macrophage-like properties, including the ability to engulf oxidized lipoproteins and transform into foam cells, further amplifying lipid deposition within the plaque. Moreover, synthetic VSMCs secrete ECM components and proteases that influence plaque stability, an imbalance that can render lesions vulnerable to rupture (93). Through this induction of VSMCs phenotypic switching, LCN-2 serves as a key mediator linking vascular inflammation to the structural remodeling that underlies atherosclerotic lesion development and complication.

#### 3.4.2 Stimulation of VSMCs proliferation and migration

LCN-2 potently stimulates VSMCs proliferation and migration, two key pathological processes that drive neointimal hyperplasia and accelerate atherosclerotic plaque progression. Under normal conditions, VSMCs in the arterial media maintain a low proliferative index and are spatially confined by the surrounding extracellular matrix. However, within the inflammatory milieu of developing atherosclerotic lesions, elevated LCN-2 acts as a direct mitogenic and chemoattractant signal for VSMCs, triggering their aberrant activation and movement (94).

LCN-2 binds to its specific receptor SLC22A17 on the VSMCs surface, activating downstream signaling cascades that promote cell cycle progression and cytoskeletal reorganization (95). This interaction leads to the phosphorylation of ERK1/2 and the activation of the PI3 K/Akt pathway. ERK signaling drives the expression of cyclin D1 and other cell cycle regulators, facilitating G1/S phase transition and enhancing VSMCs proliferation. Simultaneously, PI3 K/Akt activation promotes cell survival and stimulates the expression of MMPs, particularly MMP-2 and MMP-12, which degrade the basement membrane and perivascular extracellular matrix, thereby creating a permissive environment for VSMCs migration (96, 97).

Additionally, LCN-2 activates NF-*κ*B and ROS-dependent pathways, further amplifying the expression of migration-associated proteins such as focal adhesion kinase and integrins (98). The combined effect is a dramatic increase in VSMCs motility, enabling these cells to migrate from the tunica media into the intima, where they proliferate and contribute to the formation of the fibrous cap and the expansion of the atherosclerotic lesion. Excessive LCN-2-driven VSMCs proliferation and migration can also lead to pathological thickening of the intima and promote plaque instability, as an imbalance in VSMCs accumulation versus matrix degradation may weaken the fibrous cap. Through these concerted actions, LCN-2 serves as a critical molecular driver of the VSMCs abnormalities that underpin the structural progression and clinical complications of atherosclerosis.

#### 3.4.3 Modulation of extracellular matrix synthesis by VSMCs

LCN-2 critically modulates the synthesis of ECM components by VSMCs, thereby influencing both the structural integrity and the stability of atherosclerotic plaques. Within the arterial wall, VSMCs are the principal source of ECM, including fibrillar collagens such as Col I and Col III, elastin, proteoglycans, and glycoproteins, which collectively provide mechanical strength and resilience to the plaque (99). However, in the pro-inflammatory environment of advancing atherosclerosis, elevated LCN-2 disrupts the balanced production of these ECM constituents, leading to maladaptive matrix remodeling.

LCN-2 activates TGF-*β* signaling and the MAPK cascade, particularly the p38 and JNK pathways, which regulate the expression of ECM-related genes in VSMCs (100). Through these pathways, LCN-2 induces the transcription of Col1A1 and Col3A1, enhancing the synthesis of interstitial collagens that constitute the fibrous cap (101). Concurrently, LCN-2 up-regulates the expression of MMPs, especially MMP-2 and MMP-9, while suppressing the production of tissue inhibitors of metalloproteinases (TIMPs) (102). This protease–antiprotease imbalance shifts the ECM dynamics toward excessive degradation, undermining the tensile strength of the fibrous cap and rendering the plaque more susceptible to rupture.

Moreover, LCN-2 promotes the deposition of proteoglycans, such as versican and biglycan, which have a high affinity for retaining lipoproteins, thereby augmenting lipid accumulation within the intima (103). LCN-2 also reduces elastin synthesis, diminishing the elastic recoil capacity of the vessel wall. Through this multifaceted regulation, which includes enhancing collagen production while simultaneously driving matrix degradation and altering proteoglycan composition, LCN-2 creates a vulnerable plaque phenotype characterized by a thin, disorganized fibrous cap and a large lipid-rich necrotic core (104). Thus, LCN-2-driven modulation of VSMCs ECM synthesis represents a critical determinant of plaque progression and its clinical complications, including acute coronary syndromes.

### 3.5 LCN-2 and plaque instability and rupture

#### 3.5.1 Expansion of the lipid core

LCN-2 significantly contributes to plaque instability and the risk of rupture by driving the expansion of the lipid-rich necrotic core, a hallmark feature of vulnerable atherosclerotic lesions. The progressive enlargement of the lipid core results from an imbalance between lipid influx and efflux within the intimal microenvironment, compounded by the sustained accumulation of apoptotic and necrotic cellular debris (105). LCN-2, which is abundantly expressed in advanced plaques, promotes this pathological process through multiple interconnected mechanisms.

LCN-2 enhances the uptake of ox-LDL by macrophages while simultaneously suppressing cholesterol efflux via down-regulation of ABCA1 and ABCG1 transporters (65). This dual action leads to massive intracellular lipid accumulation and the formation of foam cells, which eventually undergo apoptosis under persistent metabolic and oxidative stress. Moreover, LCN-2 impairs the efferocytic clearance of apoptotic foam cells by reducing the expression of key phagocytic receptors such as MerTK on macrophages (106). The resulting defect in dead cell removal transforms the lesion into a pro-inflammatory state where secondary necrosis releases lipid-laden debris, further expanding the necrotic core. Besides, LCN-2 promotes the secretion of MMPs by macrophages and VSMCs, which degrade the fibrous cap and facilitate the coalescence of lipid pools (97). As the necrotic core enlarges, the overlying fibrous cap becomes thinner and more disorganized, mechanically weakening the plaque's structural integrity. Collectively, by fostering foam cell apoptosis, inhibiting efferocytosis, and amplifying matrix degradation, LCN-2 drives the relentless expansion of the lipid core, shifting the plaque toward a rupture-prone phenotype that precipitates acute cardiovascular events such as myocardial infarction and stroke.

#### 3.5.2 Thinning of the fibrous cap

LCN-2 critically promotes plaque instability and rupture by driving the thinning of the fibrous cap, a key structural determinant of atherosclerotic lesion vulnerability (107). The fibrous cap, composed primarily of Col I and Col III synthesized by VSMCs, provides mechanical strength to the plaque and separates the pro-thrombotic lipid core from the bloodstream (108). A thin, disrupted cap is the hallmark of a rupture-prone lesion. LCN-2, which is markedly elevated in advanced plaques, compromises fibrous cap integrity through multiple convergent mechanisms.

LCN-2 induces VSMCs phenotypic switching from a contractile, collagen-producing state to a synthetic, proteolytic phenotype, leading to reduced expression of Col I and Col III (99). Simultaneously, it down-regulates the synthesis of TIMPs while up-regulating the production of MMPs, particularly MMP-2, MMP-8, and MMP-9, by both VSMCs and infiltrating macrophages (97). This protease–antiprotease imbalance accelerates the degradation of existing collagen within the cap. In addition, LCN-2 promotes VSMCs apoptosis via activation of the mitochondrial death pathway and the endoplasmic reticulum stress response (109). The loss of VSMCs further depletes the cellular source of new matrix synthesis and weakens the structural framework of the fibrous cap. Furthermore, the pro-inflammatory actions of LCN-2 amplify the infiltration of activated macrophages and T lymphocytes into the shoulder regions of the plaque, where they secrete additional MMPs and inflammatory cytokines that perpetuate matrix destruction (110). As the fibrous cap progressively thins, the plaque becomes increasingly susceptible to mechanical rupture under hemodynamic stress. Through these integrated effects, including suppressing collagen production, enhancing matrix degradation, and inducing VSMCs death, LCN-2 directly contributes to fibrous cap thinning, thereby converting a stable plaque into a vulnerable lesion prone to rupture and the subsequent onset of acute coronary syndromes.

#### 3.5.3 Promotion of intraplaque hemorrhage and thrombosis

LCN-2 exacerbates plaque instability and the risk of acute thrombotic events by actively promoting intraplaque hemorrhage and thrombosis, two devastating complications of advanced atherosclerosis. Intraplaque hemorrhage typically arises from the rupture of immature, leaky neovessels that sprout into the plaque from the vasa vasorum, while thrombosis results from the exposure of highly thrombogenic plaque constituents upon fibrous cap disruption (111). LCN-2 contributes to both processes through distinct yet interconnected mechanisms.

LCN-2 drives neovascularization by stimulating the expression of VEGF and other angiogenic factors in macrophages and VSMCs (19). However, these newly formed microvessels are structurally defective, lacking adequate pericyte coverage and basement membrane integrity. LCN-2 further destabilizes these neovessels by up-regulating MMPs, which degrade the surrounding extracellular matrix, rendering the microvessels prone to leakage and rupture (97). The resulting extravasation of erythrocytes deposits free hemoglobin and iron into the plaque, amplifying oxidative stress and inflammation while expanding the lipid core. Moreover, LCN-2 directly promotes a pro-thrombotic state within the lesion. It upregulates the expression of tissue factor, the primary initiator of the coagulation cascade, on both macrophages and endothelial cells (112). This enhances the thrombogenicity of the plaque surface and the lipid-rich core. Additionally, LCN-2 activates platelets through its binding to the 24p3R receptor and via the generation of reactive oxygen species, promoting platelet aggregation and adhesion to the exposed subendothelium (113). The release of NETs induced by LCN-2 further stabilizes the thrombus by providing a scaffold rich in tissue factor and proteases.

Through these coordinated actions, including fostering leaky neovessels to trigger intraplaque hemorrhage and creating a hypercoagulable microenvironment to sustain thrombosis, LCN-2 transforms a stable plaque into a clinically dangerous lesion. This vicious cycle of hemorrhage, inflammation, and thrombosis accelerates plaque growth and instability, positioning LCN-2 as a key mediator linking vascular pathology to acute coronary syndromes and stroke, as generalized in Table 2 and Figures 2, 3.

**Table 2:** LCN-2's biological functions in regulating atherosclerosis in different cell types.

Cell type	Key effects	Major molecules/pathways	Pathological outcome	Ref.
Endothelial cells	↓ NO bioavailability, ↑ adhesion molecules, barrier disruption	eNOS, VCAM-1, ICAM-1, E-selectin, RhoA, VE-cadherin	Endothelial dysfunction, leukocyte adhesion, permeability	(48–51, 53, 54, 58)
Macrophages	M1 polarization, ↑ ox-LDL uptake, ↓ cholesterol efflux	NF-*κ*B, STAT1, CD36, SR-A, ABCA1, ABCG1, LXR	Foam cell formation, inflammatory amplification	(33, 63–66, 68, 69, 73)
VSMCs	Phenotypic switching, ↑ proliferation & migration, ECM remodeling	ERK, PI3 K/Akt, KLF4, MMP-2/9, Col I/III, TIMPs	Neointimal hyperplasia, fibrous cap thinning, plaque vulnerability	(88–92, 94, 96, 97, 102)
Neutrophils	NETosis, ROS generation, chemotaxis	PAD4, NADPH oxidase, MPO, neutrophil elastase	Inflammation, thrombosis, plaque destabilization	(32, 75, 77, 78)
T lymphocytes	Th1/Th17 polarization, Treg suppression	STAT1, STAT3, ROR*γ*t, T-bet, IFN-γ, IL-17	Adaptive immune activation, chronic inflammation	(34, 80–86)

**Figure 2:**
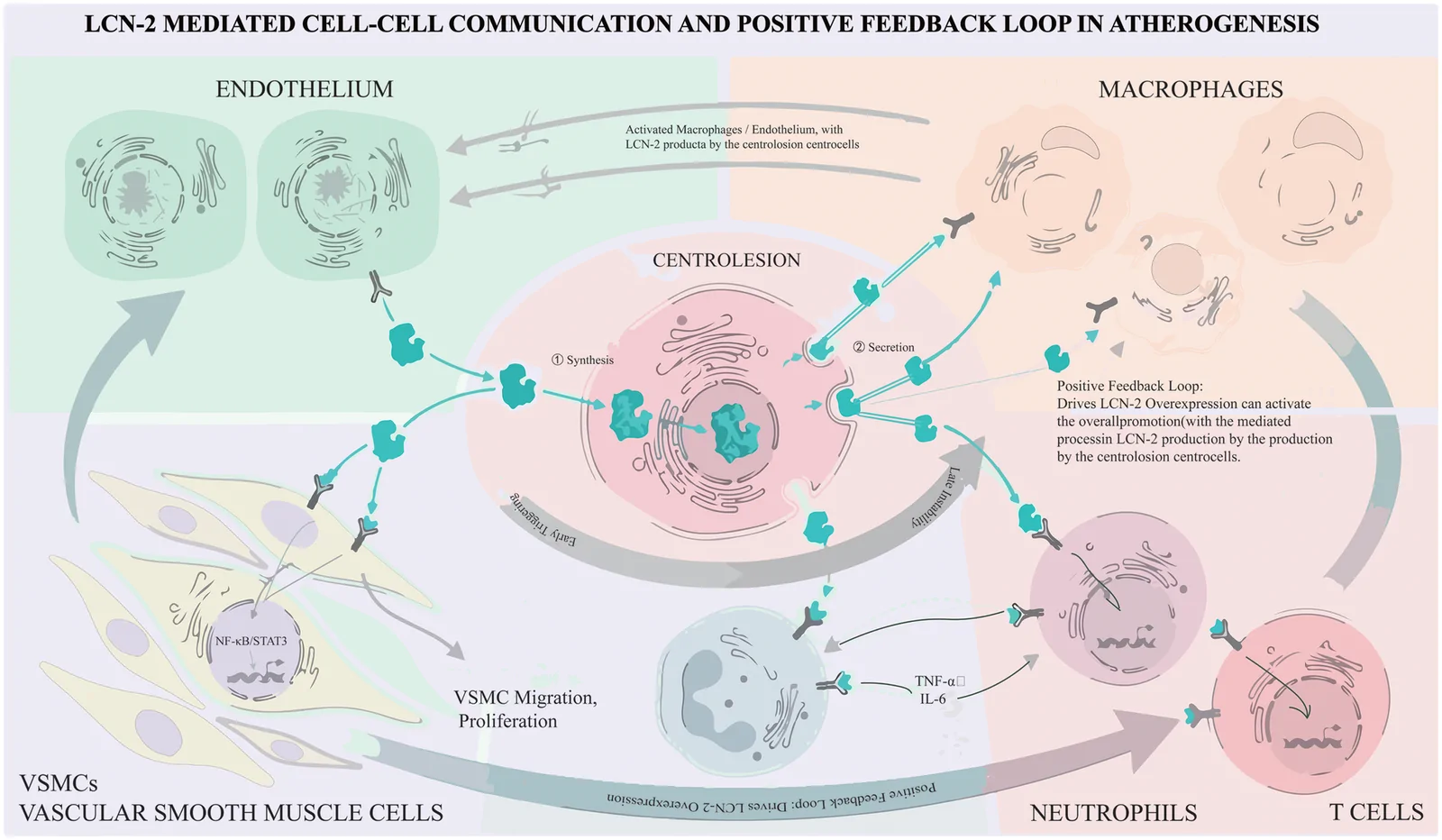
LCN-2 mediated cell-cell communication and positive feedback loop in atherogenesis.

**Figure 3:**
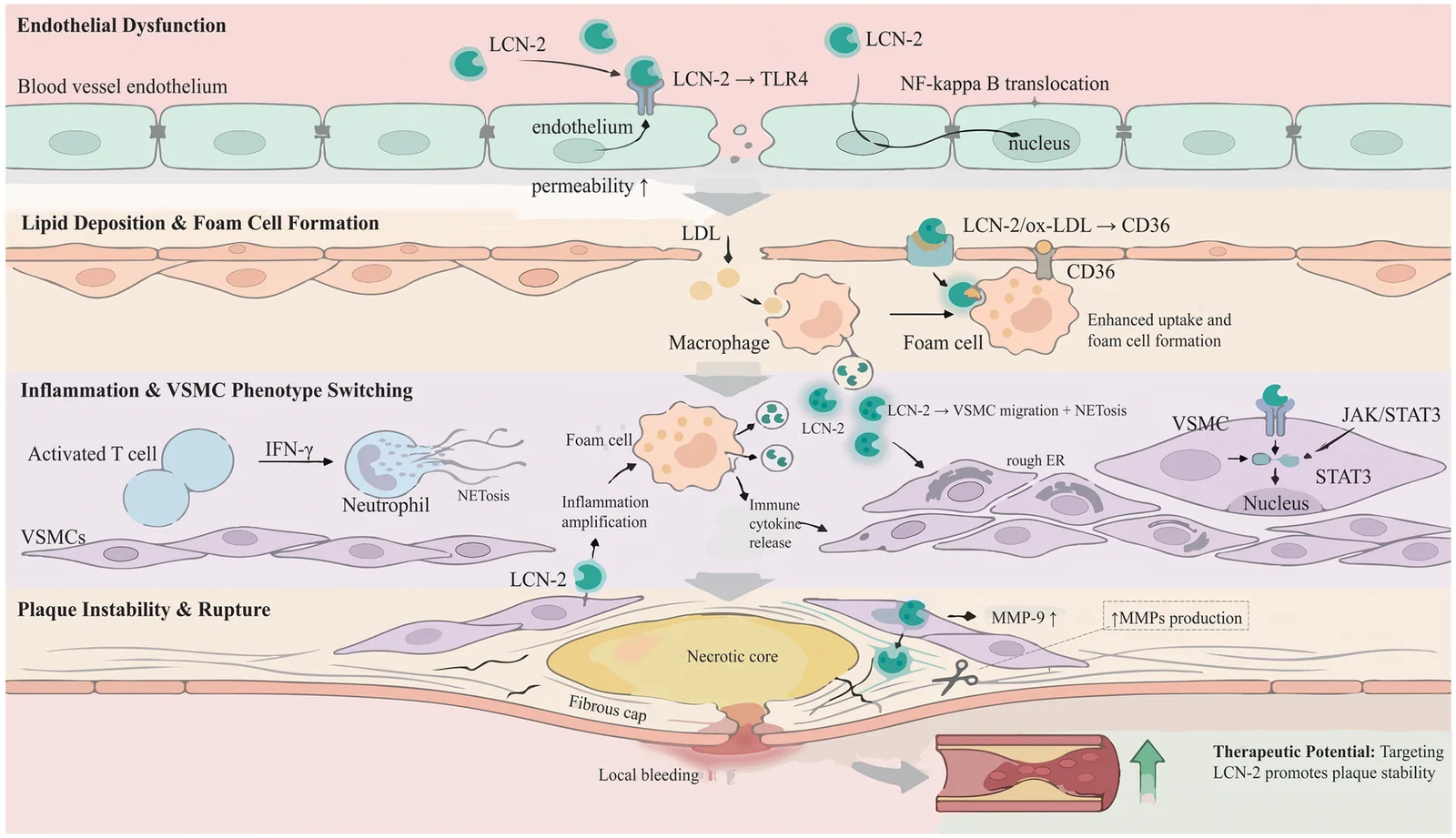
Summary of the biological functions of LCN-2 in different cell types during atherosclerosis pathogenesis.

## 4 Context-dependent dual effects of LCN-2 in atherosclerosis

The biological actions of LCN-2 in atherosclerosis are remarkably context-dependent, exhibiting paradoxical and sometimes opposing effects that are dictated by a complex interplay of factors including cell type, cellular source, disease stage, inflammatory milieu, metabolic status, and experimental model systems. This functional duality poses significant challenges for interpreting preclinical data and underscores the necessity for precise, spatiotemporally controlled investigations.

The cellular source of LCN-2 critically determines its net effect on atherosclerotic progression. Hepatocyte-derived LCN-2 has been shown to exert protective effects by promoting reverse cholesterol transport via the Nedd4-1-SR-BI axis, thereby reducing plasma lipid levels and attenuating lesion formation (62). In stark contrast, macrophage-derived LCN-2 amplifies local inflammation, enhances oxidized lipoprotein uptake, and drives foam cell formation, thereby accelerating plaque development (63). Similarly, adipocyte-secreted LCN-2 contributes to systemic insulin resistance and chronic low-grade inflammation (114), whereas neutrophil-derived LCN-2 may serve an early defensive role against bacterial pathogens but concurrently promotes NETosis and vascular injury in the established plaque (115).

The stage of atherosclerotic disease further modulates LCN-2's effects. In the early phases of atherogenesis, LCN-2 may act as a protective acute-phase responder, mobilizing innate immune defenses against microbial insults and facilitating the clearance of cellular debris (116). However, during the chronic, progressive stages of advanced atherosclerosis, sustained LCN-2 elevation becomes maladaptive, fueling persistent inflammation, promoting VSMCs phenotypic switching, and destabilizing the fibrous cap (117). This temporal dichotomy suggests that LCN-2's role transitions from a beneficial stress responder to a detrimental disease amplifier as the plaque evolves.

The inflammatory microenvironment and metabolic state also profoundly influence LCN-2 functionality. Under acute inflammatory conditions, LCN-2 can limit iron availability to pathogens and modulate excessive immune responses, potentially conferring tissue protection (118). Conversely, in the metabolically compromised milieu of obesity and diabetes, which is characterized by hyperlipidemia, hyperglycemia, and insulin resistance, LCN-2 expression is chronically upregulated and contributes to the vicious cycle of inflammation and metabolic derangement (119).

Experimental model systems introduce substantial variability in observed outcomes. Such variability stems from multiple sources, including species-specific differences between murine and human LCN-2 biology, the choice of global or cell-specific knockout approaches, and variations in dietary and genetic backgrounds among animal models, all of which may produce divergent or even contradictory findings (120). The use of acute versus chronic disease models, as well as variations in LCN-2 dosing and administration routes in interventional studies, further complicates the translational interpretation (121).

Collectively, this context-dependent duality positions LCN-2 not as a purely pro-atherogenic or anti-atherogenic molecule, but rather as a finely tuned biological rheostat whose net effect emerges from the integrated sum of its cellular origins, the local microenvironment, and the temporal stage of disease. Recognizing this complexity is essential for the rational design of therapeutic strategies that aim to harness the beneficial actions of LCN-2 while mitigating its deleterious effects in specific pathological contexts.

## 5 Potential anti-atherosclerotic strategies targeting LCN-2

Given the multifaceted role of LCN-2 in driving endothelial dysfunction, lipid deposition, inflammatory amplification, smooth muscle cell abnormalities, and plaque instability, targeting this molecule has emerged as a promising therapeutic avenue for atherosclerosis. Several preclinical strategies have been explored, ranging from genetic ablation to pharmacological inhibition and drug repurposing, each with distinct mechanisms and translational potential.

The most direct approach is LCN-2 gene knockout, which has been extensively studied in murine models. Genetic ablation of LCN-2 in ApoE^⁻/⁻^ or LDLR^⁻/⁻^ mice consistently reduces atherosclerotic lesion size and promotes features of plaque stability, such as thicker fibrous caps and smaller necrotic cores (117). While this strategy is invaluable for fundamental research, its direct clinical application is not feasible in humans. Nonetheless, these genetic studies provide strong proof-of-concept that LCN-2 is a valid therapeutic target.

A more clinically oriented strategy involves neutralizing antibodies that block LCN-2 protein activity. Systemic administration of anti-LCN-2 antibodies in preclinical models has been shown to attenuate vascular inflammation, reduce lipid deposition within plaques, and decrease the recruitment of pro-inflammatory immune cells. By sequestering circulating LCN-2, these antibodies interrupt its binding to receptors such as SLC22A17, thereby mitigating downstream signaling cascades including NF-*κ*B and MAPK pathways (122). Neutralizing antibodies represent a candidate drug class with moderate clinical translation potential, though challenges related to chronic administration, immunogenicity, and tissue penetration need to be addressed.

Small-molecule inhibitors designed to suppress LCN-2 synthesis or release offer another avenue. These compounds typically target upstream regulators of LCN-2 transcription such as NF-*κ*B, STAT3 or interfere with its secretion from activated cells (123). Preclinical studies have demonstrated that such inhibitors lower ROS generation, reduce endothelial adhesion molecule expression, and decrease macrophage foam cell formation. However, because LCN-2 regulation is embedded in complex signaling networks, small-molecule inhibitors may lack specificity and cause off-target effects, placing them at an earlier stage of drug development.

RNA interference-based approaches, including siRNA and antagomirs, silence LCN-2 mRNA expression with high sequence specificity. Local or systemic delivery of LCN-2-targeting siRNA has been shown to significantly reduce LCN-2 levels in atherosclerotic plaques and slow disease progression in animal models (124). Advances in nanoparticle encapsulation and ligand-conjugated delivery systems such as GalNAc conjugates have improved the *in vivo* stability and hepatocyte-specific targeting of these nucleic acid therapeutics (125). Although gene therapy strategies hold great promise, issues such as delivery efficiency, potential off-target silencing, and long-term safety remain to be resolved before clinical translation.

Finally, drug repurposing offers the shortest path to clinical application. Several established cardiovascular drugs, particularly statins and PPAR agonists, have been found to down-regulate LCN-2 expression as part of their pleiotropic effects. Statins reduce LCN-2 levels in both plasma and atherosclerotic lesions, correlating with decreased vascular injury and inflammation (39). PPAR*α*/*γ* agonists suppress LCN-2 transcription by binding to PPAR response elements in its promoter (126). Because these agents are already approved for other indications, their rapid translation into anti-atherosclerotic therapy targeting LCN-2 is highly feasible, though prospective clinical trials specifically evaluating LCN-2 as a pharmacodynamic marker are needed.

In summary, among the various LCN-2-targeted strategies, neutralizing antibodies, small-molecule inhibitors, and RNA-based gene therapies each present distinct translational advantages and hurdles. Neutralizing antibodies offer high target specificity and favorable pharmacokinetic profiles, making them the most clinically mature protein therapeutics. However, their chronic administration raises concerns regarding immunogenicity, high manufacturing costs, and limited tissue penetration into atherosclerotic plaques (127). Small-molecule inhibitors, in contrast, enable oral bioavailability and potentially lower production costs, yet they often lack isoform selectivity and may perturb upstream regulatory networks that govern pleiotropic cellular functions, increasing off-target toxicity risks (128). RNA interference modalities, including siRNA and antagomirs, provide exceptional sequence-based specificity and the capacity to silence LCN-2 at the transcriptional level, with recent advances in GalNAc-conjugated delivery systems enhancing hepatocyte-specific targeting and *in vivo* stability (129). Nevertheless, gene therapies face formidable translational barriers, including endosomal entrapment, potential immunostimulation, off-target silencing, and uncertain long-term safety. From a translational standpoint, antibodies and repurposed small molecules are closer to clinical application due to established regulatory pathways, whereas siRNA approaches, though promising, remain largely at the preclinical optimization stage. Ultimately, the optimal strategy may involve context-dependent selection or combinatorial regimens that leverage the strengths of each platform while mitigating their respective limitations, as generalized in Table 3 and Figure 4.

**Table 3:** Potential anti-atherosclerotic strategies targeting LCN-2.

Therapeutic Strategy	Mechanism of Action	Effect in Preclinical Models	Clinical Translation Potential	Ref.
LCN-2 gene knockout	Genetic ablation of LCN-2	↓ Lesion size, ↑ plaque stability	Fundamental research	(117)
Neutralizing antibody	Block LCN-2 protein activity	↓ Inflammation, ↓ lipid deposition	Candidate drug	(122, 127)
Small-molecule inhibitor	Suppress LCN-2 synthesis/release	↓ ROS, ↓ adhesion molecules	Drug development	(123, 128)
siRNA/antagomir	Silence LCN-2 mRNA expression	↓ LCN-2 level, ↓ AS progression	Gene therapy	(124, 125, 129)
Drug repurposing	Statins/PPAR agonists downregulate LCN-2	↓ LCN-2, ↓ vascular injury	Rapid translation	(39, 126)

**Figure 4:**
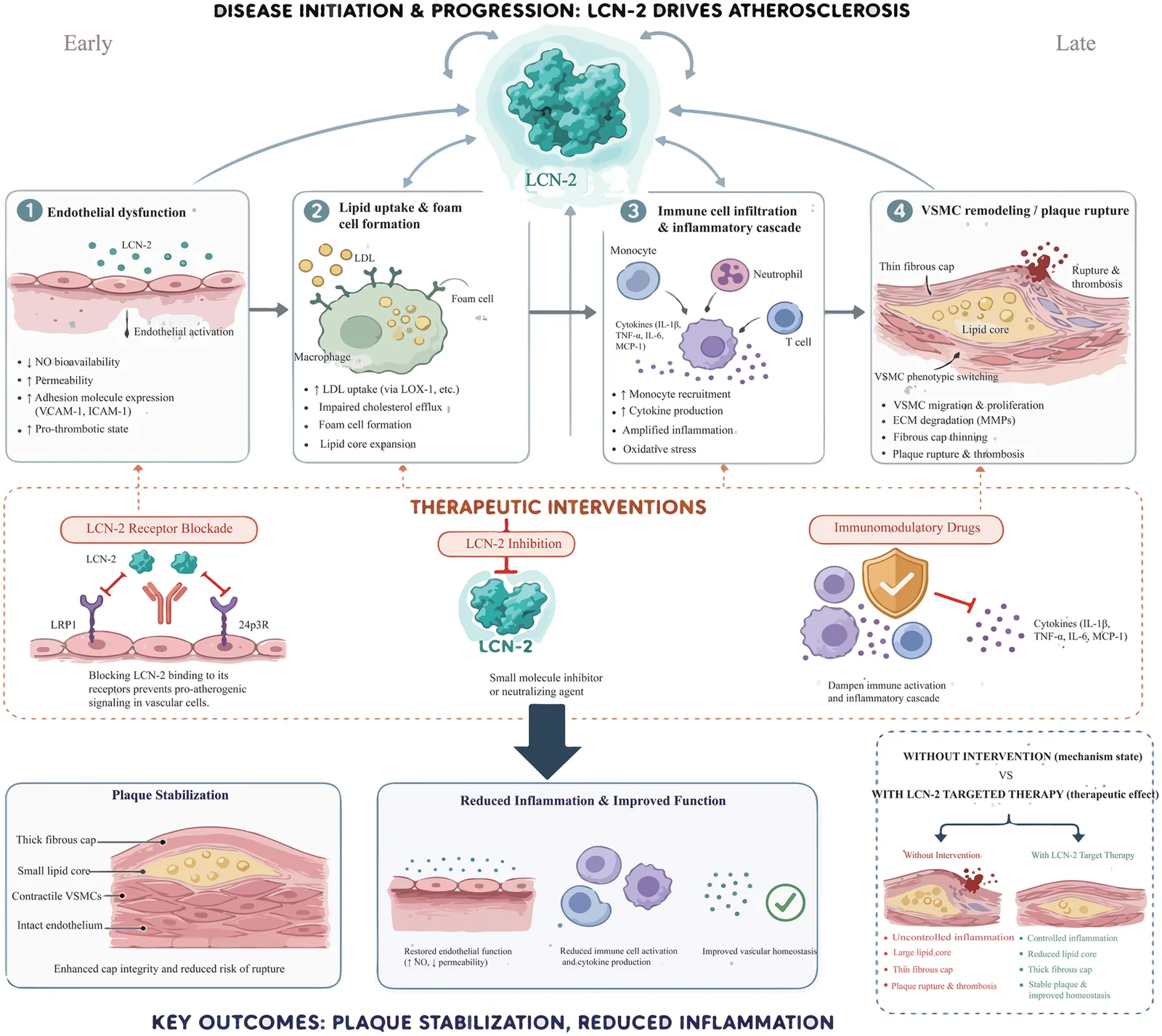
Regulatory network of LCN-2 in atherosclerosis progression and potential anti-atherosclerotic strategies targeting LCN-2.

## 6 Research limitations and future prospects

Despite growing evidence implicating LCN-2 in the pathogenesis of atherosclerosis, several critical knowledge gaps remain. While LCN-2 exerts diverse biological effects through binding to distinct ligands and receptors, the specific ligand-receptor interaction patterns and downstream signaling pathways activated by LCN-2 during different pathological stages of atherosclerosis have yet to be fully elucidated. Furthermore, the complex regulatory network linking LCN-2 to other inflammatory mediators and lipid metabolism-related molecules remains incompletely characterized. From a clinical perspective, although elevated serum LCN-2 levels have been consistently observed in patients with atherosclerosis, the diagnostic specificity of LCN-2 is limited by its susceptibility to confounding factors such as infection, renal insufficiency, and malignancy (11, 130). Moreover, the potential value of combining LCN-2 with established atherosclerotic biomarkers including hs-CRP, IL-6 and ox-LDL for improved diagnostic accuracy, as well as variations in LCN-2 expression across different atherosclerotic subtypes, has not been adequately validated in large-scale clinical studies.

We clarify that mechanistic observations from monocultured endothelial/macrophage cells represent isolated cellular responses, which may not recapitulate the complex multicellular crosstalk inside mature human atherosclerotic plaques. We explicitly note translational gaps between murine knockout/overexpression model data and human clinical atherosclerotic phenotypes, and highlight inconsistent LCN-2 activity discrepancies between rodent and human vascular tissue.

Future research should prioritize in-depth investigation of LCN-2's precise mechanisms in atherosclerosis through integrated approaches including single-cell sequencing, proteomics, and metabolomics to map its expression profiles and regulatory networks across different pathological stages and cell types such as endothelial cells, macrophages, and vascular smooth muscle cells. The application of gene editing technologies like CRISPR/Cas9 to establish cell-specific LCN-2 knockout mouse models will help clarify the distinct roles of LCN-2 derived from various cellular sources, while exploration of its specific ligands and receptors will elucidate downstream signaling pathways. In parallel, large-scale multicenter clinical studies are needed to rigorously validate the diagnostic and prognostic value of LCN-2 by correlating serum levels with atherosclerotic severity, plaque stability, and adverse cardiovascular events. Establishing combined diagnostic models incorporating other biomarkers may enhance specificity, and investigation of LCN-2 levels in non-invasive samples such as urine and saliva could facilitate broader clinical application. The development of targeted therapeutics should focus on designing specific small-molecule inhibitors or receptor antagonists guided by LCN-2 crystal structure, alongside evaluation of neutralizing antibodies and siRNA approaches in animal models. Systematic drug repurposing screens may also identify effective LCN-2 modulators with optimized administration regimens.
